# Current coronavirus (SARS-CoV-2) epidemiological, diagnostic and therapeutic approaches: An updated review until June 2020

**DOI:** 10.17179/excli2020-2554

**Published:** 2020-07-20

**Authors:** Ahmed Nabil, Koichiro Uto, Mohamed M. Elshemy, Reham Soliman, Ayman A. Hassan, Mitsuhiro Ebara, Gamal Shiha

**Affiliations:** 1 Research Center for Functional Materials, National Institute for Materials Science (NIMS), 1-1Namiki, Tsukuba, Ibaraki 305-0044, Japan; 2 Biotechnology and Life Sciences Department, Faculty of Postgraduate Studies for Advanced Sciences (PSAS), Beni-Suef University, Beni-Suef, Egypt; 3 Egyptian Liver Research Institute and Hospital (ELRIAH), Sherbin, El Mansoura, Egypt; 4 Faculty of Science, Menoufia University, Menoufia, Egypt; 5 Tropical Medicine Department, Faculty of Medicine, Port Said University, Egypt; 6 Graduate School of Pure and Applied Sciences, University of Tsukuba, 1-1-1 Tennodai, Tsukuba, Ibaraki 305-8577, Japan; 7 Graduate School of Industrial Science and Technology, Tokyo University of Science, 6-3-1 Niijuku, Katsushika-ku, Tokyo 125-8585, Japan; 8 Hepatology and Gastroenterology Unit, Internal Medicine Department, Faculty of Medicine, Mansoura University, Egypt

**Keywords:** COVID-19, SARS-CoV-2, Remdesivir, Diagnosis, Epidemiology, Therapy

## Abstract

Coronaviruses are a group of enveloped viruses with non-segmented, single-stranded, and positive-sense RNA genomes. In December 2019, an outbreak of coronavirus disease 2019 (COVID-19) caused by the novel severe acute respiratory syndrome coronavirus 2 (SARS-CoV-2), in Wuhan City, China. The World Health Organization (WHO) declared the coronavirus outbreak as a global pandemic in March 2020. Fever, dry cough and fatigue are found in the vast majority of all COVID-19 cases. Early diagnosis, treatment and future prevention are keys to COVID-19 management. Currently, the unmet need to develop cost-effective point-of-contact test kits and efficient laboratory techniques for confirmation of COVID-19 infection has powered a new frontier of diagnostic innovation. No proven effective therapies or vaccines for SARS-CoV-2 currently exist. The rapidly increasing research regarding COVID-19 virology provides a significant number of potential drug targets. Remdesivir may be the most promising therapy up till now. On May 1, 2020, Gilead Sciences, announced that the U.S. Food and Drug Administration (FDA) has granted emergency use authorization (EUA) for the investigational Remdesivir as a potential antiviral for COVID-19 treatment. On May 7, 2020, Gilead Sciences, announced that the Japanese Ministry of Health, Labour and Welfare (MHLW) has granted regulatory approval of Veklury® (Remdesivir) as a treatment for SARS-CoV-2 infection, the virus that causes COVID-19 acute respiratory syndrome, under an exceptional approval pathway. Also, Corticosteroids are recommended for severe cases only to suppress the immune response and reduce symptoms, but not for mild and moderate patients where they are associated with a high-risk side effect. Based on the currently published evidence, we tried to highlight different diagnostic approaches, side effects and therapeutic agents that could help physicians in the frontlines.

## Introduction

In December 2019, a novel coronavirus, SARS-CoV-2, was identified as the pathogen causing coronavirus disease (COVID-19) in Wuhan, China. On March 11, 2020, the World Health Organization declared COVID-19 as a global pandemic (Whitworth, 2020[71]).

COVID-19 is an enveloped, positive-sense, single-stranded RNA virus that belongs to the beta-CoV genus, which also includes SARS-CoV and MERS-CoV. It shares 89 % nucleotide identity with bat SARS-like CoVZXC21 and 82 % identity with human SARS-CoV (Chan et al., 2020[15]).

COVID-19 is transmitted by inhalation or contact with infected droplets. The incubation period for COVID-19 is on average, 5-6 days, but can be up to 14 days. During this period, also known as the “presymptomatic” period, some infected persons can be contagious, from 1-3 days before symptom onset (Wei et al., 2020[69]). The clinical manifestations of COVID-19 varied from asymptomatic carrier status, acute respiratory disease (ARD) and pneumonia. The prevalence of asymptomatic cases is significant (20-86 % of all infections) and is defined as individuals with positive viral nucleic acid tests but without any COVID-19 symptoms. Most people with COVID-19 develop only mild (40 %) or moderate (40 %) disease, approximately 15 % develop a severe disease that requires hospitalization and oxygen support, and 5 % have a critical disease with complications such as respiratory failure, acute respiratory distress syndrome (ARDS), sepsis and septic shock, thromboembolism, and/or multiorgan failure, including acute kidney injury and cardiac injury (CDC, 2020[14]) Older age, co-morbidities such as diabetes, hypertension, cardiac disease, chronic lung disease, cancer and BMI > 40 kg/m^2^ have been reported as risk factors for severe disease and death (CDC, 2020[13]).

## Common Signs and Symptoms

Wang and colleagues (2020[66]) reported that there are 6 common signs and symptoms that 30 % of the patients have felt including fever (98.5 %), fatigue (69.9 %), dry cough (59.4 %), anorexia (39.8 %), myalgia (34.8 %), dyspnea (31.1 %) and for the most common comorbidities are hypertension (31.1 %) and cardiovascular disease (14.5 %). Symptoms may develop 2 days to 2 weeks following exposure to the virus (CDC, 2020[14]). According to Wu and McGoogan (2020[74]), among 72,314 SARS-CoV-2 cases reported to the Chinese Center for Disease Control and Prevention (CCDC), 81 % were mild (mild or absent pneumonia), 14 % were severe (dyspnea, hypoxia, > 50 % lung involvement within 1-2 days), 5 % were critical (respiratory failure, shock, multiorgan dysfunction), and 2.3 % were fatal. Symptoms in children with infection appear to be uncommon, although some children with severe COVID-19 have been reported (CDC, 2020[13]). Based on currently available information and clinical expertise, risk factors for severe COVID-19 include older adults ≥ 65 years as well as people of all ages with chronic lung disease or moderate to severe asthma, serious heart conditions, diabetes, severe obesity, chronic kidney disease, liver disease and immunocompromised people (CDC, 2020[13]).

## Suggested Infection Mechanism

Upon infection with COVID-19, it binds to the host cell's angiotensin-converting enzyme 2 (ACE2) receptors which commonly expressed on the epithelial cells of alveoli, trachea, bronchi, and bronchial serous glands of the respiratory tract. Then the virus enters and replicates in these cells (Liu et al., 2011[45]). The newly developed virions are then released and infect new target cells. Unfortunately, there is no specific antiviral treatment or vaccine recommended for COVID-19 that is currently available.

## Current Epidemiological Situation

**Figure 1:**
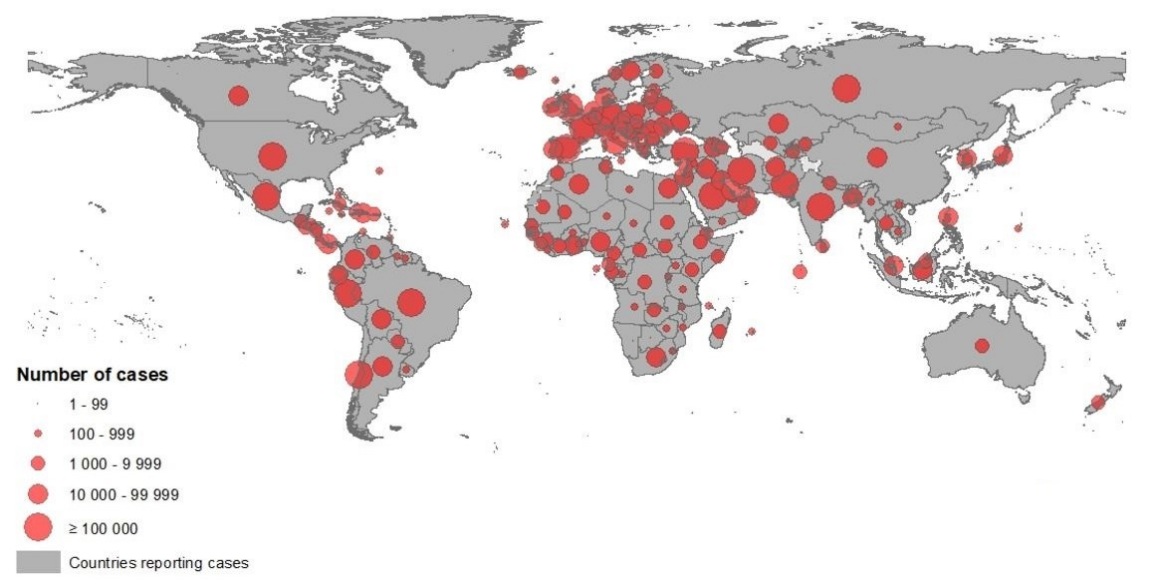
Novel coronavirus COVID-19 geographical distribution over the word 2020-05-09 (ECDC, 2020)

According to the European Centre for Disease Prevention and Control (ECDC), since 31 December 2019 and as of 14 June 2020, 7,759,691 cases of COVID-19 have been reported including, most cases in America (n = 3788548) were reported from the United States (2,074,526), Brazil (850,514) and Peru (225,132), followed by Europe (n = 2,170,600): most cases were reported in Russia (520,129), United Kingdom (294,375) and Spain (243,605), Asia (n = 1557541): most cases were in India (320,922), Iran (184,955) and Turkey (176,677), Africa (n = 233528): most cases were in South Africa (65,736), Egypt (42,980), Nigeria (15,682), Oceania (n = 8766): most cases were in Australia (7,290), New Zealand (1,154) and Guam (185) (Figure 1(Fig. 1)), including 430,127 deaths, most deaths in America (n = 201,874) were reported from the United States (115,436), Brazil (42,720) and Mexico (16,872), followed by Europe (n = 182674): most deaths were in United Kingdom (41,662), Italy (34,301) and France (29,398), Asia (n = 39147): most deaths were in India (9,195), Iran (8,730) and Turkey (4,792), Africa (n = 6294): most deaths were in Egypt (1,484), South Africa (1,423) and Algeria (760), Oceania (n = 131): most deaths were in Australia (102), New Zealand (22) and Guam (5) (ECDC, 2020[26]).

**Figure 2:**
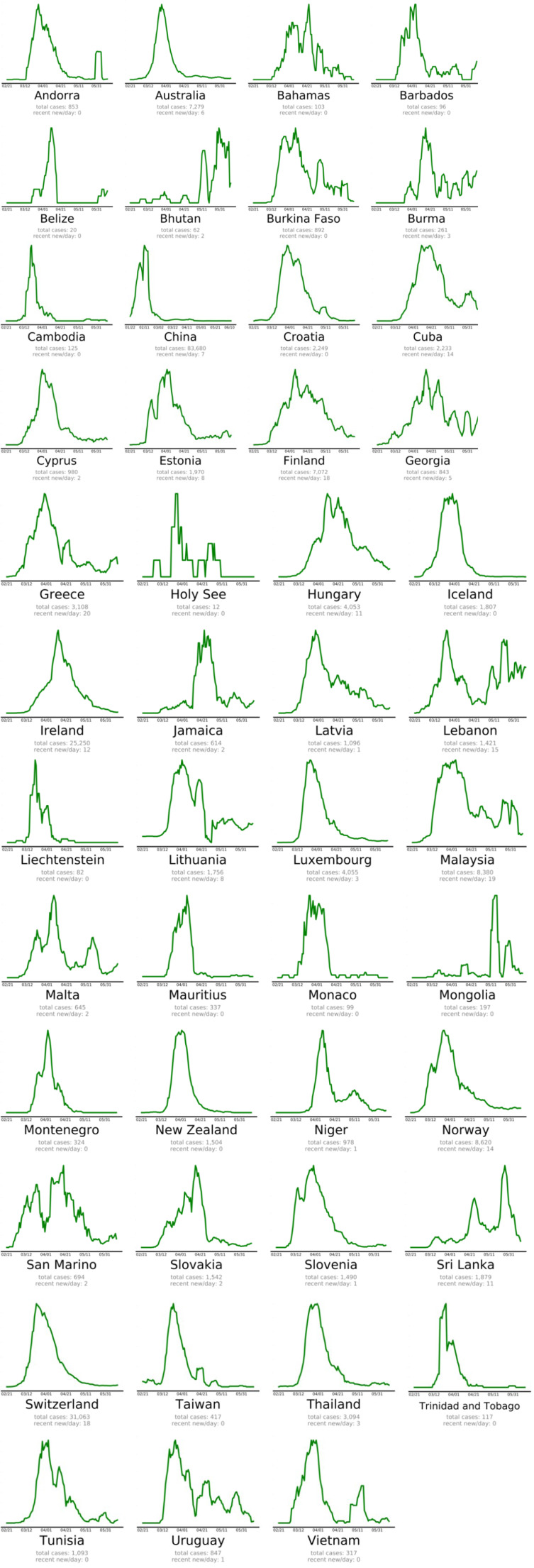
Countries beating COVID-19 in alphabetical order (EndCoronavirus, 2020)

**Figure 3:**
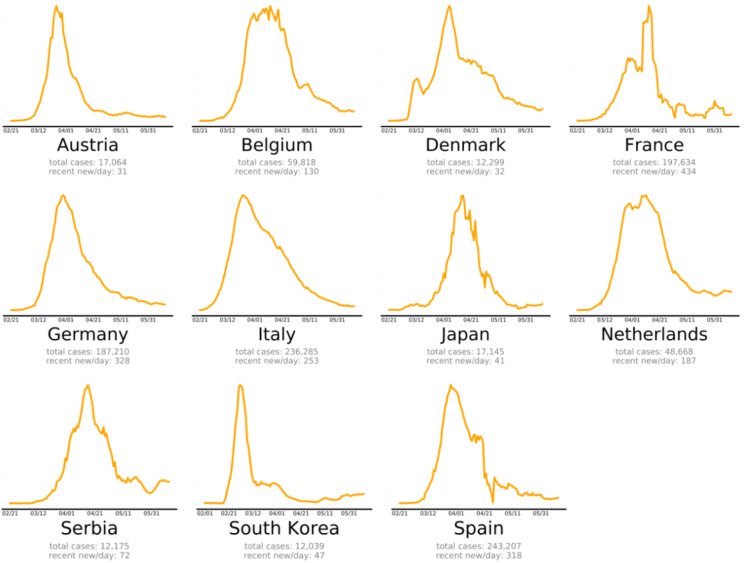
Countries that are nearly there (EndCoronavirus, 2020)

**Figure 4:**
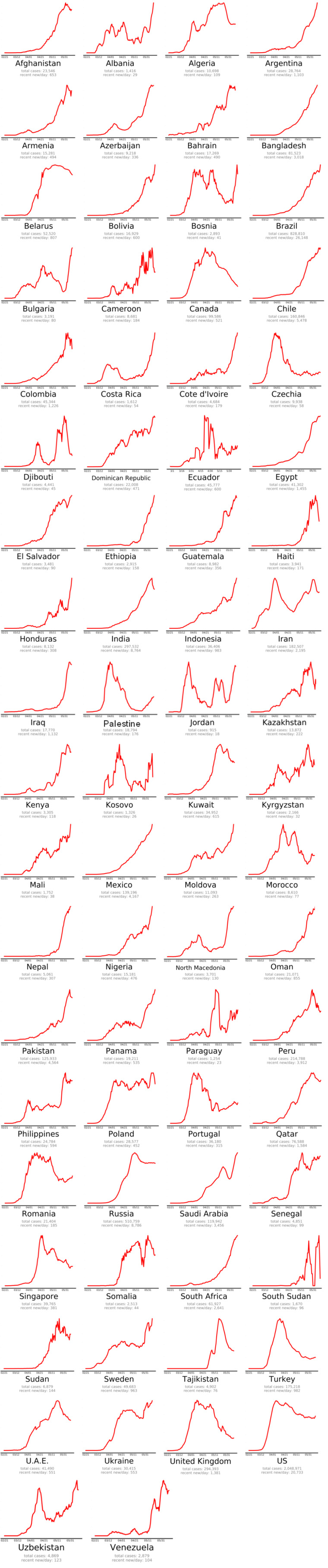
Countries that need to do an action (EndCoronavirus, 2020)

The countries that beat COVID-19 were divided into three groups as follows: countries beating COVID-19: green plots (Figure 2(Fig. 2)), countries that are nearly there: yellow plots (Figure 3(Fig. 3)) and countries that need to take action: red plots (Figure 4(Fig. 4)). These plots adjusted for each country to better show the data. The vertical axis is plotted in arbitrary units, to easily compare the shapes of the curves (EndCoronavirus, 2020[30]).

## SARS-CoV-2 Diagnosis

The diagnosis of COVID-19 mainly depends on the demonstration of the virus in respiratory secretions by special molecular tests. Common laboratory findings include normal/ low white cell counts with elevated C-reactive protein (CRP). The computerized tomographic chest scan is usually abnormal even in those with no symptoms or mild disease (Singhal, 2020[58]). In addition to laboratory testing capacity and reagent shortages, the rapidly growing SARS CoV 2 pandemic has encouraged many diagnostic manufacturers to develop and sell fast and easy-to-use equipment to facilitate testing outside the laboratory (WHO, 2020[72]).

**Figure 5:**
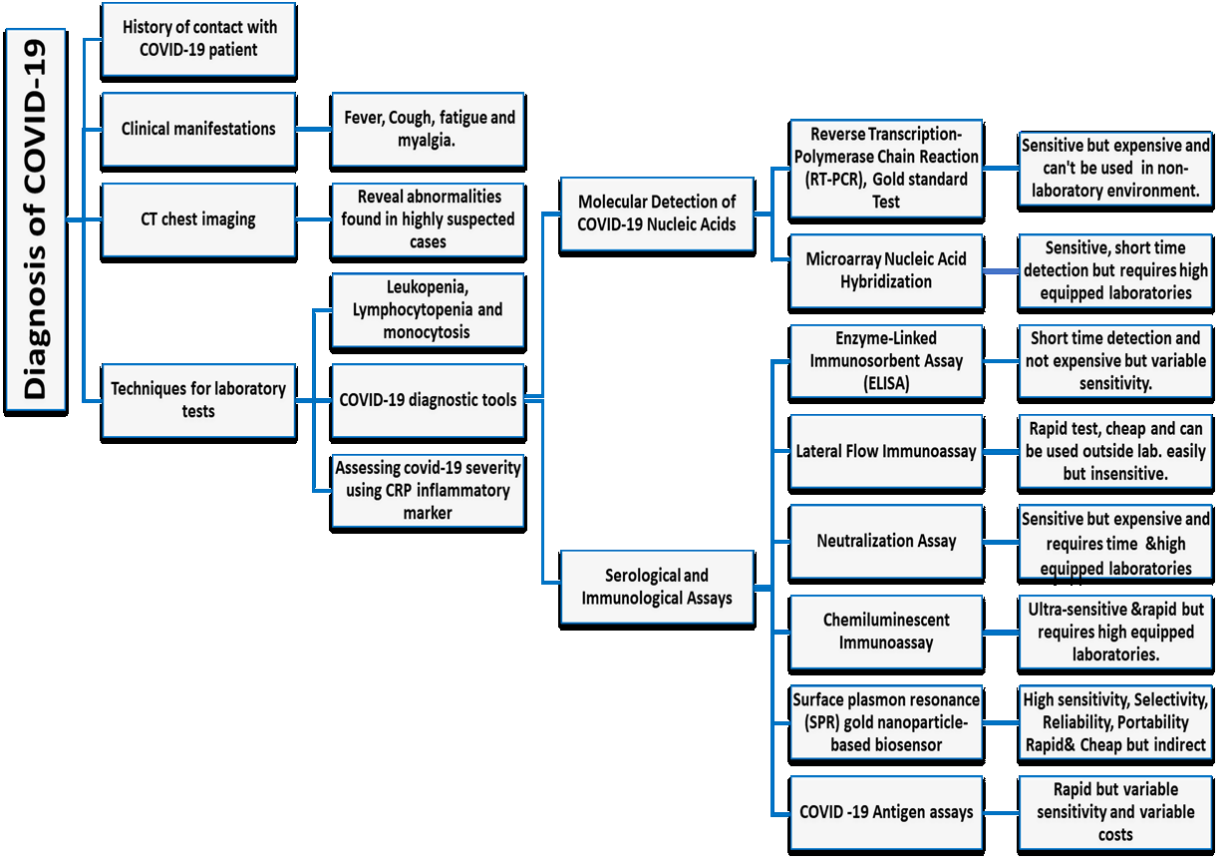
The Diagnostic Model for COVID-19

Currently, there are two main categories commercially available for COVID-19 tests. The first category includes molecular assays for detection of SARS-CoV-2 viral RNA using polymerase chain reaction (PCR)-based methods. The second category includes serological and immunological assays that largely depend on detecting antibodies produced by individuals as a result of exposure to the virus or on the detection of antigenic proteins in infected individuals. It is necessary to ensure that these two categories of tests serve overlapping purposes in the management of the SARS-CoV-2 pandemic (Carter et al., 2020[11]). Current COVID-19 diagnostic tools and techniques are shown in Table 1(Tab. 1) (References in Table 1: Cai et al., 2020[7]; Carter et al., 2020[11]; Chan et al., 2020[16]; Chen et al., 2010[19]; Park et al., 2009[52]; Wang et al., 2005[65]; Whiteman et al., 2018[70]) and a diagnostic model for COVID-19 in Figure 5(Fig. 5).

## SARS-CoV-2 Different Therapeutic Approaches

Symptomatic treatment and oxygen therapies represent the major treatment interventions for patients with severe infection. Mechanical ventilation may be necessary in cases of respiratory failure refractory to oxygen therapy, whereas hemodynamic support is essential for managing septic shock (Cascella et al., 2020[12]). 

To the best of our knowledge, different therapeutic approaches have been evaluated against COVID-19 *in vivo, vitro* and in clinical trials. Many of these therapies had a great impact on clinical recovery. Current COVID-19 therapies are shown in Table 2(Tab. 2) (References in Table 2: Baron et al., 2020[3]; Caly et al., 2020[8]; Cao et al., 2020[10]; Chang et al., 2020[17]; Chen et al., 2020[18]; clinicaltrials.gov, 2020[20]; Deng et al., 2020[22][23]; Devaux et al., 2020[24]; Du and Chen, 2020[25]; Elfiky, 2020[28]; Fintelman-Rodrigues et al., 2020[32]; Gao et al., 2020[34]; Graci and Cameron, 2006[36]; Hadadi et al., 2020[37]; Leng et al., 2020[41]; Li et al., 2020[42]; Liang et al., 2020[43]; Lim et al., 2020[44]; Meng et al., 2020[48]; Richardson et al., 2020[53]; Runfeng et al., 2020[54]; Russell et al., 2020[55]; Sanders et al., 2020[56]; Sheahan et al., 2020[57]; Sun et al., 2020[59][60]; Tang et al., 2020[61]; Wang et al., 2020[67]; Xu et al., 2020[75][76]; Zhou et al., 2015[80]).

## SARS-CoV-2 Therapeutic Approaches - Side Effects

Despite the approved beneficial effects of these therapeutic approaches, recent studies concluded that most of these candidate's administration has a toxic effect in overdoses, causing common and severe adverse effects including nausea, pruritus, arrhythmias, hypoglycemia, anemia, jaundice, hyperlipidemia, electrolyte abnormalities, acute renal injury, hematological disorders, hyperuricemia, neuropsychiatric effects and various drug-drug interactions.

Chloroquine (CQ) interferes with ventricular repolarization that increases the risk of torsades de pointes (TdP) and may cause sudden cardiac death (Ursing et al., 2020[64]), also it causes neuropsychiatric manifestations including confusion agitation, psychosis, mania, hallucinations, paranoia, suicidal ideation, depression, insomnia and catatonia (Aneja et al., 2019[2]) as well as severe hypoglycemia (El-Solia et al., 2018[29]). Moreover, CQ has severe immunological mediated adverse effects including drug reaction with eosinophilia and systemic symptoms (DRESS) (Girijala et al., 2019[35]), Stevens-Johnson syndrome (Leckie and Rees, 2002[40]) and toxic epidermal necrolysis (Cameron et al., 2014[9]).

Lopinavir/Ritonavir (LPV/r) combination has been reported to have gastrointestinal disorders, so in some SARS-CoV-2 patients, the treatment was stopped due to these severe side events (Owa and Owa, 2020[51]). Notwithstanding the minimal side effects of Teicoplanin, it may cause thrombocytopenia in some treated cases (Terol et al., 1993[63]).

A recent clinical trial regarding Remdesivir with severe COVID-19 patients concluded that adverse events including hypokalemia, constipation, hypoalbuminemia, anemia, jaundice, hyperlipidemia, liver enzyme elevation and thrombocytopenia were reported (Wang et al., 2020[68]). 

An exploratory randomized controlled trial assessing the efficacy and safety of Arbidol in COVID-19 patients reported that patients had adverse events including diarrhea, nausea and loss of appetite (Eikenberry et al., 2020[27]), also hypotension, acute renal injury, teratogenicity, hypersensitivity, electrolyte abnormalities, fatigue, diarrhea, weakness, anemia and chest pain are the most common risk factors during treatment of COVID-19 patients using inhibitors of the renin-angiotensin system (Ingraham et al., 2020[38]).

Zhang and colleagues (2020[79]) reported that intravenous transplantation of Wharton's jelly derived mesenchymal stem cells (hWJCs) was safe and effective especially, in COVID-19 critical severe cases. Regarding Tocilizumab that was used as a treatment for severe COVID-19 cases, it may cause serious adverse reactions, like intestinal perforation, candidiasis and lipid metabolism abnormalities (Tao et al., 2020[62]). 

FDA has approved convalescent plasma therapy in COVID-19 critical patients, but up till now, only three studies with small sample size reported effectiveness and safety so more clinical trials are needed to ensure both safety and efficacy (Bloch et al., 2020[6]).

Otherwise Direct-acting antivirals (DAAs) demonstrated, a safe therapeutic approach with common side effects including fatigue, headache, nausea and neuropsychiatric symptoms (Medeiros et al., 2017[47]). Concerning using of Favipiravir (Avigan®) as a treatment for COVID-19 patients, it was reported that Favipiravir elevates plasma uric acid, so this finding should be considered in hyperuricemia, gout and kidney impairment patients (Mishima et al., 2020[49]). 

Despite the beneficial effect of Corticosteroids with COVID-19 patients, they are associated with a high risk of death, side effects like bacterial infections and hypokalemia so they are not recommended for mild and moderate COVID-19 patients, but they should be used in severe cases only to suppress the immune response and reduce symptoms (Yang et al., 2020[77]).

## Chloroquine Triggers Oxidation and Hemolytic Anemia in G6PD Deficient Cases & World Health Organization Discontinued its Treatment Trials

Glucose-6-phosphate dehydrogenase (G6PD) deficiency is one of the most common human enzymatic disorders affecting around 400 million people worldwide (Luzzatto and Arese, 2018[46]). Decreased G6PD production results in low levels of NADPH and reduced glutathione stimulating hemolytic anemia which is characterized by oxidative stress and red blood cell lysis (Francis et al., 2013[33]). 

The risk of hemolytic anemia should be considered during Chloroquine/Hydroxy Chloroquine (CQ/HCQ) therapy of patients with G6PD deficiency (Mohammad et al., 2018[50]).

Beauverd and colleagues (2020[4]) reported that SARS-CoV-2 infection can enhance severe acute hemolysis in patients with G6PD‐deficiency, and CQ/HCQ can worsen this crisis. During the treatment of SARS-CoV-2, it is important to carefully monitor potential hemolytic effects of CQ/HCQ in G6PD deficiency cases. If a decline in hemoglobin levels during the first days of CQ/HCQ treatment is observed, the treatment should be stopped. Hemolysis usually is reversible after finishing therapy with CQ/HCQ (De Franceschi et al., 2020[21]). Also, Kapoor and Kapoor (2020[39]) warned of the use of CQ because of the risk of hematological disorders in patients with G6PD deficiency.

In contrast, both (Youngster et al. 2010[78]; Beutler 1994[5]) concluded that CQ or HCQ mono-therapies are safe also in G6PD deficient cases.

Afra and colleagues (2020[1]) reported that infections might be the most common causes of hemolysis in G6PD deficient patients. Thus, SARS‐CoV‐2 patients may show significant hemolysis even before CQ or HCQ administration.

Finally, SARS‐CoV‐2 treatment using CQ or HCQ, especially in areas with high G6PD deficiency prevalence, should alert medical staff to this possible harmful effect. The US Food and Drug Administration warned of cardiotoxicity caused by hydroxychloroquine and mentioned G6PD as a baseline test before the onset of hydroxychloroquine treatment (FDA, 2020[31]). Moreover, in July 2020 the WHO discontinued clinical trials with hydroxychloroquine and lopinavir/ritonavir treatment arms for COVID-19 (WHO, 2020[73]), where both therapies produced little and no reduction in the mortality of hospitalized SARS‐CoV‐2 cases when compared to standard of care.

## Conclusion

Finally, COVID-19 pandemic is a highly infectious disease caused by the novel coronavirus SARS-CoV-2 that can be transmitted through droplets and close contact and represents a global public health crisis. Fever, fatigue and dry coughs are the most common signs and symptoms of COVID-19. Due to rapid transmission, countries around the world should increase attention to disease surveillance systems. SPR gold nanoparticle-based biosensors may be a promising diagnostic technique as it had high sensitivity, selectivity, reliability, portability, is rapid and cheap, but this method is an indirect method, where it detects antibody, so developing of SPR biosensor to detect COVID-19 itself still is a great challenge. No proven effective therapies or vaccines for SARS-CoV-2 currently exist. The most promising therapy up till now maybe Remdesivir, also we recommend Corticosteroids therapy for severe cases only to suppress the immune response and reduce symptoms, but not for mild and moderate patients where they are associated with high-risk side effects. G6PD should be considered as a baseline test for starting CQ or HCQ treatment protocol to avoid its possible hemolytic effect. We should further strive to develop specific medications, support the research and development of vaccines, and also decrease morbidity and death of SARS-CoV-2 to preserve the population.

## Notes

Ahmed Nabil, Mitsuhiro Ebara (Research Center for Functional Materials, National Institute for Materials Science (NIMS), 1-1Namiki, Tsukuba, Ibaraki 305-0044, Japan; Tel: 008180-6661-5342, E-mail: EBARA.Mitsuhiro@nims.go.jp) and Gamal Shiha (Egyptian Liver Research Institute and Hospital (ELRIAH), Sherbin, El Mansoura, Egypt; Hepatology and Gastroenterology Unit, Internal Medicine Department, Faculty of Medicine, Mansoura University, Egypt; Tel: (+20)1223280501, E-mail: g_shiha@hotmail.com) equally contributed as corresponding authors. 

## Authors contribution

Ahmed Nabil: Resources, Conceptualization, Original draft writing, Supervision, Review & Editing. Koichiro Uto: Original draft writing, Review & Editing. Mohamed M. Elshemy: Original draft writing, Review, Editing & Resources. Reham Soliman: Writing, Review & Editing. Ayman A. Hassan: Writing & Editing. Mitsuhiro Ebara: Conceptualization, Resources, Original draft writing, Supervision, Review & Editing. Gamal Shiha: Conceptualization, Original draft writing, Review, Editing & Supervision.

## Acknowledgement

All authors express their great gratitude for researchers, physicians, nurses, health care technicians and all co-workers in the frontlines in Egypt, Japan and any spot of the globe who spend their life during fighting this virus, hoping this work could help them in their mission.

Special thanks for Ebara Labo., NIMS, Japan research team & ELRIAH, El Mansoura, Egypt researchers, physicians, nurses and health care technicians.

## Conflict of interest

The authors declare that they have no conflict of interest.

## Figures and Tables

**Table 1:**
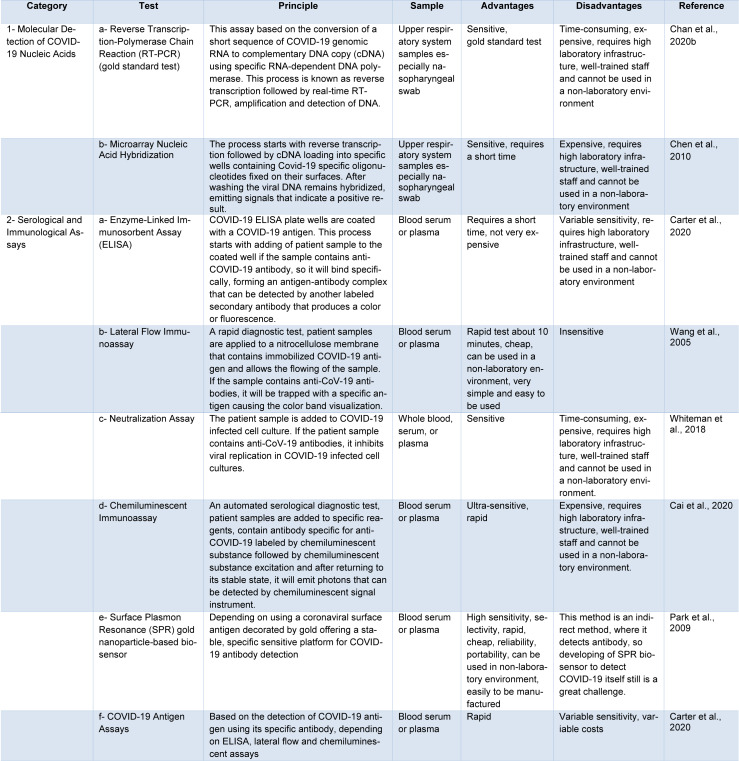
Current SARS-CoV-2 diagnostic tools and techniques

**Table 2:**
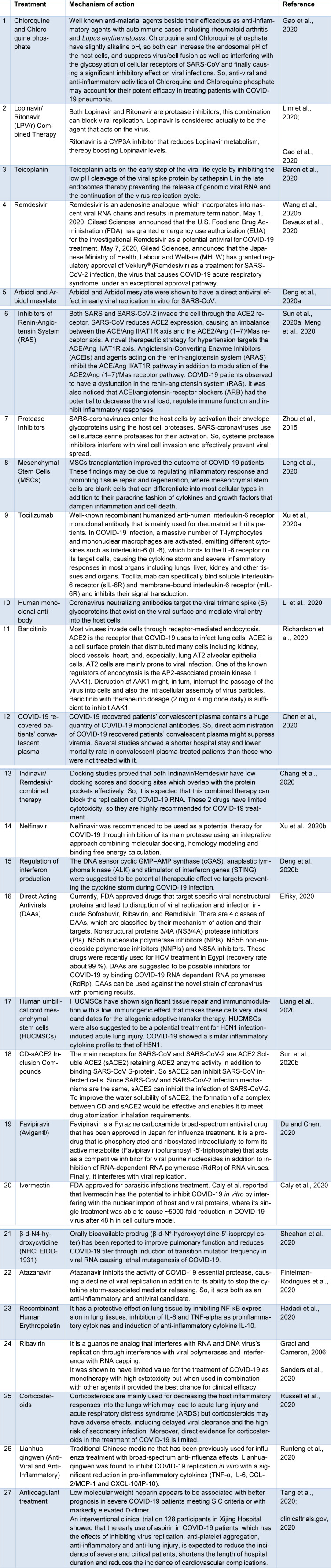
Different SARS-CoV-2 therapeutic approaches and mechanisms
